# Establishment and characterisation of primary human oesophageal organoids as a versatile epithelial model

**DOI:** 10.1007/s00383-026-06704-z

**Published:** 2026-11-13

**Authors:** Giada Benedetti, Paola Bonfanti, Paolo De Coppi, Giovanni Giuseppe Giobbe

**Affiliations:** 1 Stem Cells and Regenerative Medicine Section, Developmental Biology and Cancer, Great Ormond Street Institute of Child Health, University College London, London, UK; 2 Epithelial Stem Cell Biology and Regenerative Medicine Laboratory, The Francis Crick Institute, London, UK; 3 Institute of Immunity and Transplantation, Division of Infection and Immunity, University College London, Pears Building, London, UK; 4 Department of Specialist Neonatal and Paediatric Surgery, Great Ormond Street Hospital, London, UK

**Keywords:** Oesophageal organoids, Paediatric oesophagus, Epithelial modelling

## Abstract

**Purpose:**

Human patient-derived oesophageal epithelial models are limited, and organoid cultures often have short passaging lifespans, restricting studies of tissue homeostasis and disease. We aimed to establish and characterise primary oesophageal organoids from non-pathologic epithelium as a renewable epithelial source and adaptable modelling platform.

**Methods:**

Primary oesophageal tissues were obtained from endoscopic biopsies and surgical resections. Basal epithelial cells were isolated and cultured under optimised conditions to establish long-term three-dimensional organoids. Organoid-derived cells were expanded as two-dimensional monolayers and re-seeded into 3D culture. Epithelial identity and differentiation were assessed by immunostaining and RT-qPCR (reverse transcription-quantitative polymerase chain reaction) analysis of markers.

**Results:**

Primary organoids were established and maintained while preserving epithelial markers. Expression of KRT14, involucrin, loricrin and filaggrin confirmed the oesophageal phenotype. Larger, multilayered primary organoids showed increased expression of differentiation-associated markers relative to 2D monolayers and re-seeded small organoids, indicating culture configuration influences epithelial differentiation while maintaining lineage identity.

**Conclusions:**

We established a robust and reproducible protocol for generating and maintaining oesophageal organoids across culture formats. These models retained epithelial features while exhibiting culture-dependent differentiation. This platform enables complementary 2D and 3D investigation of oesophageal epithelial biology and supports future comparison of healthy and disease-derived tissues for patient-specific modelling and mechanistic studies.

**Supplementary Information:**

The online version contains supplementary material available at https://doi.org/10.1007/s00383-026-06704-z.

## Introduction

The oesophagus is a muscular foregut tube that connects the pharynx to the stomach and transports food by coordinated peristalsis and sphincter control [1–3]. It comprises mucosa, submucosa, muscularis propria and adventitia [1]. For epithelial modelling, the mucosa is the key compartment: it consists of a non-keratinised stratified squamous epithelium, lamina propria and muscularis mucosae, arranged in longitudinal folds that flatten on distension [4]. Submucosal glands open onto the luminal surface and secrete mucus and bicarbonate to lubricate the mucosa and contribute to local pH regulation. Their ducts traverse the submucosa and epithelium before reaching the lumen [5, 6]. The epithelium provides a barrier against mechanical abrasion during bolus transit and is organised along papillae [1, 7]. At the gastro-oesophageal junction, squamous mucosa abruptly transitions to gastric columnar epithelium [8].

Human oesophageal epithelium, comprising approximately 20-30 cell layers with papillary folding, is thicker and more complex than its murine counterpart, which is thinner, keratinised and lacks papillae and extensive submucosal glands [9]. Epithelial cells form a continuum from basal to early, mid and late suprabasal layers, defined by keratin and differentiation markers. Basal cells express KRT14/15, P63 and related basement-membrane-associated proteins, while differentiating cells switch to KRT4/13 and later produce involucrin, loricrin and filaggrin [6, 7, 10]. Continuous basal proliferation renews the epithelium approximately every 11 days [6]. Although mouse lineage-tracing supports a single progenitor pool that balances proliferation and differentiation [11, 12], human studies indicate greater basal heterogeneity, including proliferative and quiescent subpopulations [10, 13–15]. Submucosal gland ducts may also provide a latent progenitor reservoir after injury [6]. Epithelial maintenance depends on niche signalling: BMP (bone morphogenetic protein) promotes differentiation and is restrained by Noggin to preserve progenitor activity, whereas Wnt appears comparatively dispensable for oesophageal progenitor maintenance and organoid formation [16]. These cues are directly relevant when recreating the niche for in vitro expansion.

Organoid culture now provides a practical platform to interrogate this epithelium. Human pluripotent stem cells can be directed to oesophageal progenitors by stepwise foregut patterning, with BMP/TGFβ (transforming growth factor beta) inhibition favouring oesophageal over respiratory fate [9, 17, 18]. The resulting progenitors generate stratified squamous epithelium in three-dimensional organoids. Expansion is supported by factors such as EGF (epidermal growth factor) and FGF10 (fibroblast growth factor 10). Primary patient-derived oesophageal organoids can likewise be established from healthy biopsies and display outer basal-like cells with inner differentiation, including involucrin expression [10, 19]. However, existing primary cultures generally have a limited passaging lifespan, and there is still no consensus on the molecular inputs required to recreate niche conditions for stable long-term culture [20]. Applications remain comparatively few, although organoids have already been used to study Notch signalling in eosinophilic oesophagitis and to model epithelial development and stratification [17–19]. Against this background, the present study aimed to establish and characterise primary human oesophageal organoids from healthy tissue as a renewable epithelial model that can be adapted across complementary culture formats.

## Methods

### Ethics

All paediatric tissues were donated with informed consent from their guardian, obtained by an independent research coordinator under an approved research licence. Human paediatric biopsies were collected and stored in compliance with the Human Tissue Act under NHS licence 18DS02. Samples, sourced from various procedures at Great Ormond Street Hospital NHS Foundation Trust, were used to derive organoid lines.

### Cell derivation

The oesophageal mucosa protocol was optimised from the gastric gland isolation protocol [21, 22]. Tissues were collected in ice-cold Advanced DMEM/F12 medium (Thermo, 12634) supplemented with 10 mM HEPES (Thermo, 15630080), 2 mM Glutamax (Thermo, 35050061), and 1% penicillin-streptomycin (Thermo, 15140122), referred to as “ADMEM+++”, and processed immediately after collection. Alternatively, they were collected in dry gauze and immediately transferred to ADMEM+++ upon arrival.

Endoscopic biopsies (3 mm diameter) were processed without further dissection, while larger resection specimens were cut open to expose the mucosa as a flat layer, and dissociated from muscle layers (Supplementary Table 1).

The tissue was cut into small pieces (< 1 mm diameter) with a scalpel and transferred to a 15 mL tube. Pieces were washed vigorously with 10 mL fresh Hanks’ Balanced Salt Solution (HBSS; Gibco, 12082739) using a 10 mL pipette pre-coated with 1% bovine serum albumin (BSA; Sigma-Aldrich, A9418) in Dulbecco’s phosphate-buffered saline without calcium and magnesium ions (DPBS; Sigma-Aldrich, D8537). After washing, the pieces were allowed to settle, and the supernatant was discarded.

The mucosal pieces were incubated in a chelating buffer prepared in-house consisting of 5.6 mmol/L Na2HPO4, 8.0 mmol/L KH2PO4, 96.2 mmol/L NaCl, 1.6 mmol/L KCl, 43.4 mmol/L sucrose, 54.9 mmol/L D-sorbitol, 0.5 mmol/L DL-dithiothreitol, and 8 mM ethylenediaminetetraacetic acid (EDTA) (all Sigma-Aldrich; EDTA, E8008) in Milli-Q water.

Oesophageal samples were incubated for 15 min at 37 °C on a planar shaking platform. After incubation, EDTA was discarded, and the mucosal pieces were resuspended in 3 mL of TrypLE Express (Thermo, 12605010) for 10 min at 37 °C. The samples were vigorously pipetted with a BSA-coated tip, vortexed for 3 min. ADMEM+++ was then added up to 8 mL for shaking. After straining through a 70 μm cell strainer (Appleton Woods, 352350), cells were centrifuged at 200 *g* for 5 min at 4 °C.

The supernatant was aspirated, and the pellet was resuspended in ice-cold liquid Matrigel^®^ Growth Factor Reduced (MGFR; Corning, 354230). Droplets of 30 µL were aliquoted into pre-warmed multi-well plates and incubated inverted for 20 min at 37 °C to induce gelation. OE (oesophageal organoid) complete medium (Table 1) with 10 µM Rho kinase inhibitor Y-27,632 (ROCKi, Tocris, 1254) was added (1 mL for 12-well, 500 µL for 24-well) and changed every 3 days.

**Table 1:**
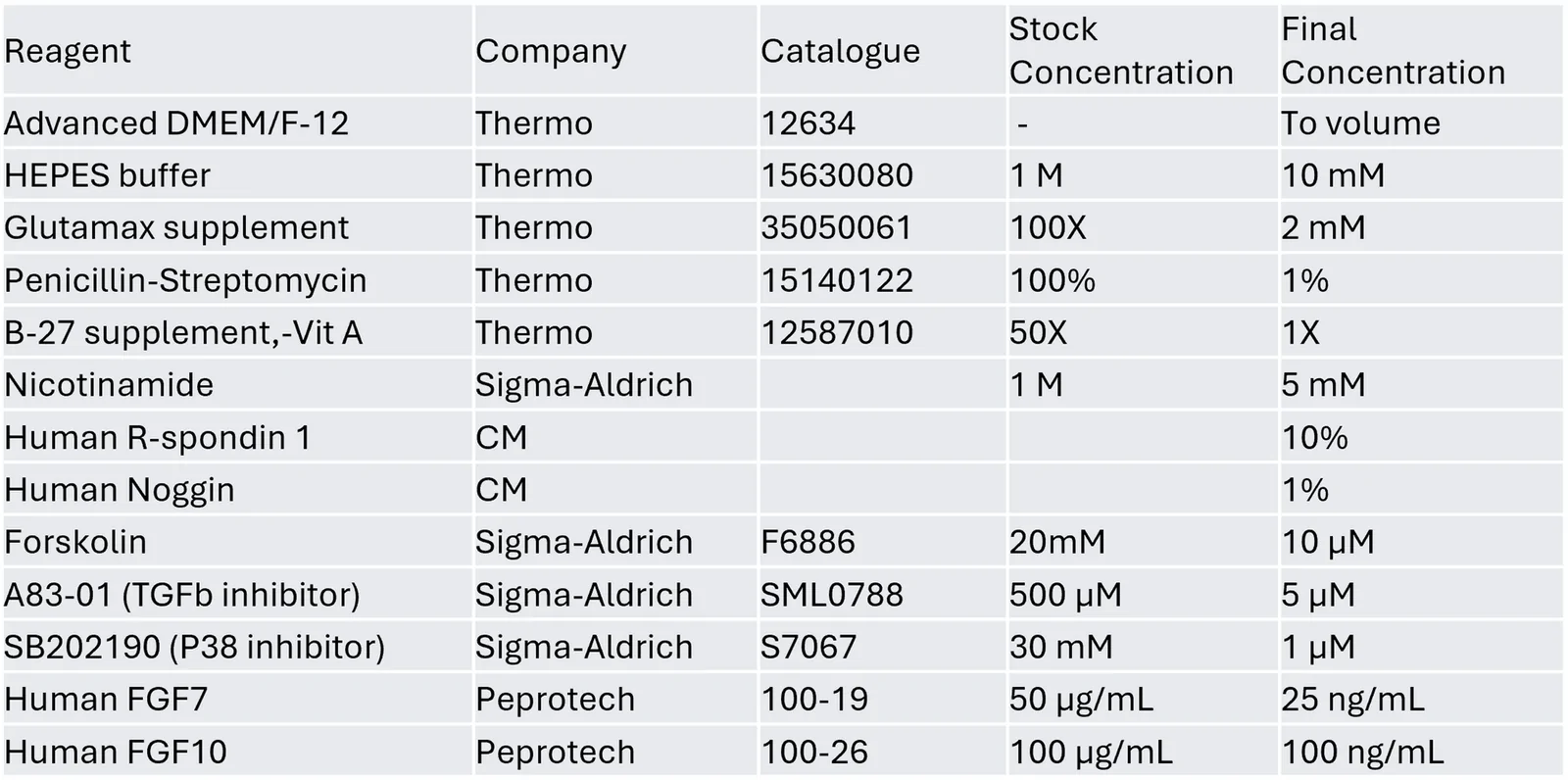
Composition of OE complete medium adapted from Busslinger et al [10]

### Cell splitting

After retrieval and centrifugation, the organoid pellet was resuspended in 1 mL of TrypLE Express, incubated at 37 °C for 10 min, and dissociated with a P1000 tip. TrypLE was quenched with 10 mL ice-cold ADMEM+++ and centrifuged at 300 *g* for 5 min at 4 °C. After supernatant removal, organoids were resuspended in MGFR in the appropriate volume, incubated for gelation for 15 min at 37 °C, and then OE complete medium with ROCKi was added.

Bright-field images of organoid cultures were acquired using a Zeiss Axio Observer A1 inverted widefield microscope with a Colibri 5 LED light source and an Axiocam MRm camera.

### 2D monolayers

2D monolayers were obtained either by outgrowth from mucosa pieces or from organoids released from MGFR. Both cell sources were allowed to attach to collagen I-coated plates (50 µg/mL collagen I (FirstLink, 60-35-810) in PBS, coated for 30 min at 37 °C, then washed with DMEM) and cultured in OE medium. Once established, monolayers were split by removal of medium, PBS washing, and incubation with TrypLE Express at 37 °C until visible detachment. Detached cells were harvested into a 15 mL tube, brought to 10 mL with DMEM, and centrifuged. The resulting cells could either be seeded in MGFR or reseeded in 2D culture.

### Immunostaining

Organoids released from MGFR or coverslips were fixed in 4% PFA (paraformaldehyde; Sigma-Aldrich) for 20 min at RT (room temperature) with rotation. After washing, residual PFA was quenched with 0.1 M NH_4_Cl (Sigma-Aldrich) for 1 h. Blocking and permeabilization were then carried out for 1 h at RT with Triton X-100 (Sigma-Aldrich, T9284) in 1% BSA. Primary antibodies were incubated for 24 h at 4 °C, followed by washing and overnight incubation with secondary antibodies (Table 2). Finally, unbound secondary antibodies were washed. Organoids were imaged in a drop of Fluoromount (Sigma-Aldrich, F4680) covered with a coverslip on a FluoroDish (WPI, FD35-100). Coverslips were mounted on slides with Fluoromount and acquired with a Zeiss Axio Observer A1 with a Colibri 7 LED light source.

**Table 2:**
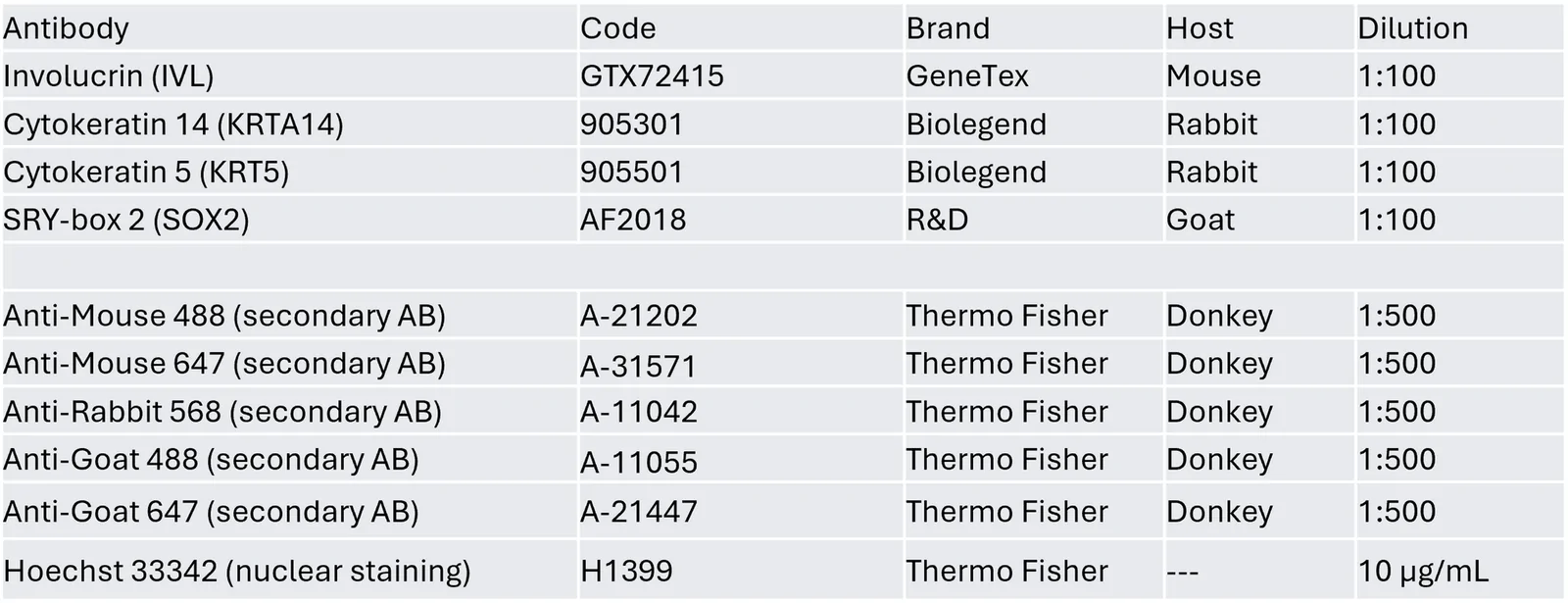
Primary and secondary antibodies used for immunostaining

### RNA extraction

RNA was extracted using the RNeasy Mini kit (Qiagen, 74104), following the manufacturer’s instructions. Organoids in MGFR were released from MGFR, washed, and transferred to the lysis buffer provided with the kit. RNA was eluted with the nuclease-free water provided with the kit, quantified by spectrophotometry, and either immediately reverse transcribed to cDNA or stored at −80 °C.

### Reverse transcription

Stored RNA was thawed on ice. Reverse transcription was performed with the High-Capacity cDNA Reverse Transcription Kit (Applied Biosystems, 4368814) according to the manufacturer’s instructions. cDNA was then immediately used for RT-qPCR or stored at −20 °C.

### RT-qPCR

RT-qPCR was conducted using custom Thermo Fisher primers (Table 3) and Universal SYBR Green Master Rox (Merck, 4913850001). The reactions were performed on a QuantStudio 5, results analysed with QuantStudio Design & Analysis Software. *GAPDH (glyceraldehyde-3-phosphate dehydrogenase)* expression normalised Ct (cycle threshold) values were presented as relative fold change to controls (matched by patient), using the ΔΔCt method, and shown as mean ± SD.

**Table 3:**
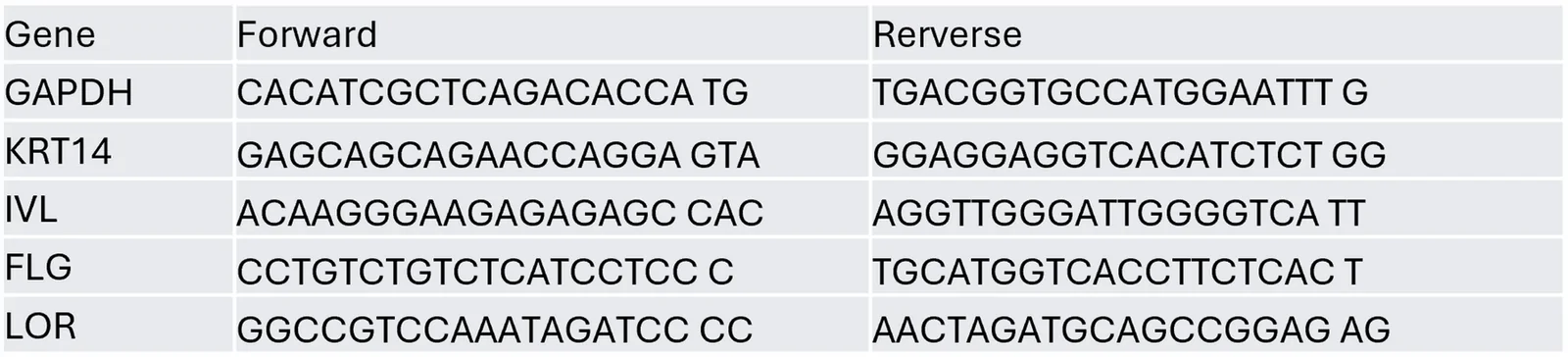
Custom Thermo Fisher probes used for RT-qPCR.

## Results

### Organoids characterisation

**Fig. 1:**
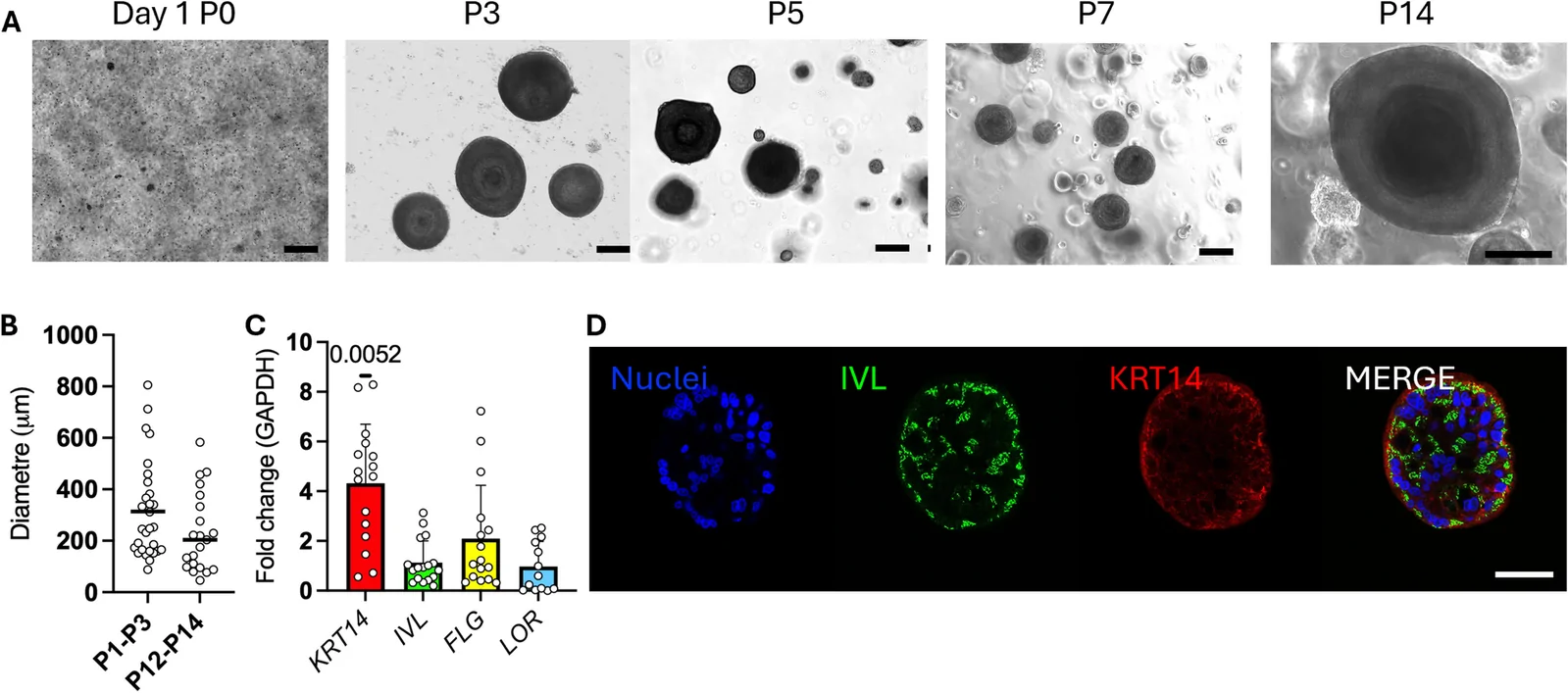
**A** Bright-field images of five oesophageal organoid lines derivation at different passages. Scale bar 200 μm **B** Diameter measurement (µm) of oesophageal organoids at early and later passages. Single data points represent single organoids from 5 lines, mean represented as bars. T-test not significant. **C** RT-qPCR results analysing 4 target genes (KRT14, IVL, FLG, LOR) in organoids normalised to matching tissue. *N* = 7 for tissue, *N* = 16 for organoids over 8 patients. Mann-Whitney U Test tissue vs. organoids **D** Immunofluorescence showing paediatric oesophagus organoids stained for Nuclei in blue (Hoechst), involucrin (IVL) in green, cytokeratin 14 (KRT14) in red. Scale bar 100 μm

The optimised protocol for cell isolation and medium composition allowed derivation of oesophageal organoids. From day 0 of culture, progenitor cells were identified within MGFR droplets, with presence of debris representing residual ECM from the derivation. Upon splitting, organoid lines were established, with dense organoids reflecting internal stratification of cell layers (Fig. 1A). Eight organoid lines were obtained and used in following analysis.

The organoids exhibited large dimensions from early passages (P1-P3), with diameters ranging considerably, from 150 to 800 μm. Organoid lines were stably passaged up to passage 14, and diameter remained stable across passages (Fig. 1B). RT-qPCR analysis of oesophageal epithelium markers revealed that, when normalised to corresponding tissue samples, the organoids were predominantly composed of basal stem cells and early differentiating cells. Among the markers analysed, only *KRT14* (*cytokeratin 14*, a basal-cell marker) showed significantly higher expression than native tissue, suggesting a relatively greater abundance of basal cells (Fig. 1C). *IVL*,* FLG *and* LOR* (*involucrin*, *filaggrin* and *loricrin*, markers of more differentiated layers) did not differ significantly. Immunofluorescence characterisation of the organoids confirmed the absence of a lumen, and an organisation characterised by an external layer of KRT14-positive cells, and inner cells positive for IVL (Fig. 1D).

### 2D cultures derivation

**Fig. 2:**
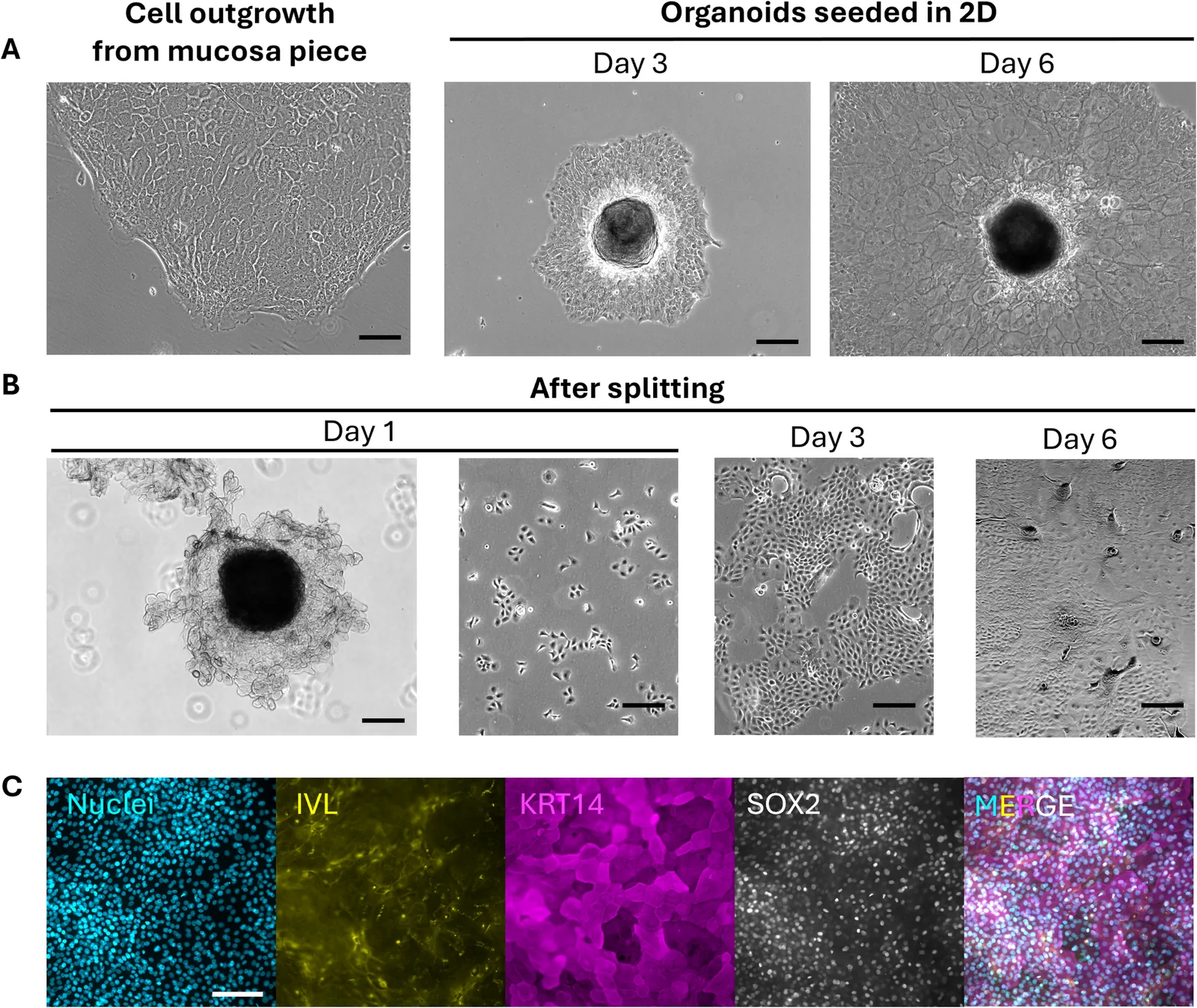
**A** Bright-field images of oesophageal monolayer cultures on collagen-coated wells. Left panel: cell outgrowth from a mucosa piece. Right panel: cell outgrowth from organoid, day 3 and 6 after seeding. Scale bar 200 μm **B** Bright-field images of oesophageal monolayer cultures on collagen-coated wells after splitting of the monolayer. Day 1: organoid that generated the monolayer found floating upon splitting, and single cells adhered. Days 3 and 6: cell growth in monolayer. Scale bar 200 μm **C** Immunofluorescence showing oesophageal monolayers stained for Nuclei in cyan (Hoechst), involucrin (IVL) in yellow, cytokeratin 14 (KRT14) in magenta, and SRY-box 2 (SOX2) in grey. Scale bar 100 μm

The optimised media conditions also supported the 2D growth of epithelial monolayers. When seeded onto collagen I-coated wells or flasks, cells were able to either outgrow directly from mucosa tissue pieces or sprout from an established organoid (Fig. 2A). Upon passaging, the monolayers could be easily dissociated and reseeded as single cells, successfully forming a new monolayer. On the other hand, organoids used to generate the monolayer remained floating, eventually died, and were removed from the culture (Fig. 2B). They could be passaged for up to 10 passages, after which growth rate declined.

Characterisation of the 2D cultures revealed strong SOX2 expression, indicative of epithelial progenitor identity. Consistently with features of basal epithelial identity, cells were highly positive for KRT14, while IVL was poorly expressed (Fig. 2C).

### Organoids vs. 2D cultures comparison

**Fig. 3:**
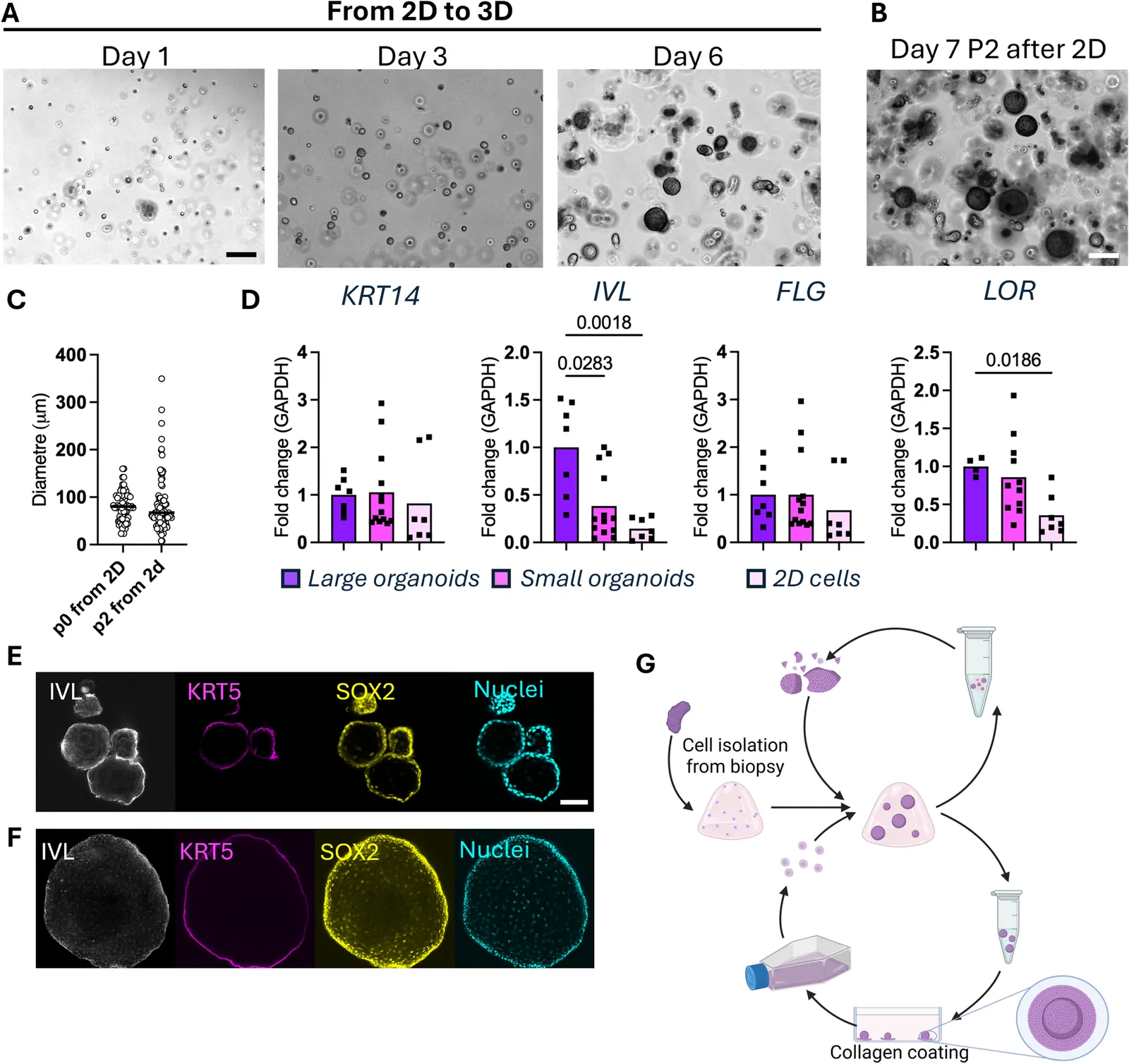
**A** Bright-field images of single cells from a 2D monolayer seeded in MGFR throughout one week of culture. Scale bar 200 μm **B** Bright-field images of oesophageal organoids maintained for 2 passages in 3D culture after 2D culture. Scale bar 200 μm **C** Diameter measure (µm) of oesophageal organoids at P0 after moving from 2D to 3D culture and at P2 after moving from 2D to 3D culture. Single data points represent single organoids from 2 lines, mean represented as bars. T-test not significant. **D** RT-qPCR results analysing 4 target genes (KRT14, IVL, FLG, LOR) in large or small organoids and 2D monolayers, normalised to large organoids. *N* > 7 (6 patients). Kruskal Wallis test **E**,** F** Immunostaining of small organoids (**E**), and large organoids (**F**), showing nuclei in cyan, SRY-box 2 (SOX2) in yellow, cytokeratin 5 (KRT5) in magenta, and involucrin (IVL) in white. Scale bar 50 μm. **G** Scheme of the available culture strategies of oesophageal epithelial cells. Biorender.com

Interestingly, upon dissociation of the monolayers into single cells and reseeding in MGFR, the cells reorganised and formed organoids within 7 days (Fig. 3A). These organoids were smaller than those in the original cultures, but could be further propagated in 3D (Fig. 3B). Larger organoids started emerging after two passages (Fig. 3C).

Gene expression was compared across the original 3D organoid cultures (large organoids), the smaller organoids derived from 3D reseeding of monolayer single cells (small organoids), and the 2D cultures. Large organoids exhibited similar levels of the basal marker *KRT14* to those observed in small organoids and monolayer cultures. However, they showed increased expression of differentiation-associated markers, with a statistically significant upregulation of *IVL*. The differentiation marker *FLG* was expressed at similar levels among the conditions, while *LOR* had a significant reduction in the monolayer compared to larger organoids (Fig. 3D). Monolayers and small organoids displayed more comparable expression profiles, suggesting a similar cellular state.

From an immunofluorescence perspective, organoids of both sizes were strongly positive for SOX2. KRT5 expression was localised at the outer cell layers, with IVL expressed in different patterns (Fig. 3E, F).

It can be concluded that these epithelial cells are very versatile. Following initial organoid derivation in MGFR, cells can be maintained in 3D cultures, adapted to 2D monolayer conditions, and subsequently re-established as organoids (Fig. 3G). This bidirectional transition between culture systems reflects the ability of these cells to preserve a basal-like progenitor state across conditions, underpinning their capacity for self-renewal and reorganisation.

## Discussion

The present study demonstrates the successful generation of primary oesophageal organoids from human-derived tissue. These organoids provide a physiologically relevant platform to model epithelial biology and can be maintained in culture for longer periods under defined conditions that carefully balance proliferation and differentiation signals. Oesophageal organoids offer an important opportunity to bridge basic research and clinical paediatric surgery, particularly in understanding oesophageal atresia (OA), tracheo-oesophageal fistula (TOF), epithelial injury and stricture formation. Their greatest translational potential lies in disease mechanism and regenerative medicine studies. Derived from adult stem cells or pluripotent stem cells, they reproduce key aspects of oesophageal epithelial development, differentiation and repair, providing a human in-vitro model to investigate developmental signalling pathways, disease mechanisms and responses to therapeutic interventions [18]. For paediatric surgeons, they could help clarify the biology underlying congenital oesophageal malformations and identify new strategies to improve healing and reduce postoperative complications [23]. Organoid-derived epithelial cells provide a renewable source of patient-specific cells to seed decellularised or synthetic scaffolds, supporting the development of engineered oesophageal substitutes for challenging conditions such as long-gap OA. Combining these cells with myogenic progenitors, fibroblasts and, eventually, vascular and neural components may help generate more functional constructs. Although significant challenges remain in reproducing the full-thickness anatomy, mechanics and peristalsis of the oesophagus, organoids offer a valuable platform to optimise cell-scaffold interactions and test candidate constructs before preclinical studies. 

Maintenance of the oesophageal epithelium in vitro required tight modulation of key signalling pathways. In particular, low and controlled Wnt activity was necessary to preserve squamous identity [9]. Suppression of BMP and TGF-β signalling (achieved through Noggin and TGF-β inhibitors) prevented premature differentiation and sustained the basal progenitor population [17]. Additionally, inhibition of the p38 MAPK (mitogen-activated protein kinase) pathway reduced stress-induced differentiation and supported continued proliferation [24]. Together, these conditions enabled stable expansion while maintaining tissue-specific identity. Oesophageal organoids were also used to generate two-dimensional monolayers, which provide a simplified system for controlled experimental manipulation. Interestingly, the growth required collagen coating of the plate surface. This matches the fact that quiescent basal cells of the oesophagus have been reported to produce collagen that favours attachment to the basal lamina [10]. However, consistent with their reduced structural complexity, monolayers showed lower expression of differentiation markers compared to three-dimensional cultures. Indeed, organoid size emerged as a key determinant of differentiation status: larger organoids showed higher expression of selected differentiation markers relative to smaller organoids and monolayers, suggesting a more mature epithelial architecture. Conversely, smaller organoids retained a more progenitor-like phenotype. This was consistent with the absence of stratification, that is, where the differentiating process occurs.

Overall, this work establishes a versatile primary human oesophageal epithelial platform that supports long-term 3D organoid culture and reversible transition to 2D monolayers, providing a foundation for future mechanistic and disease-modelling studies.

## Supplementary Information

Below is the link to the electronic supplementary material.

Supplementary Material 1

## Data Availability

Data supporting the findings of this study are available within the paper and its Supplementary Information or available upon request.
