# Supplementary material for: Establishment and characterisation of primary human oesophageal organoids as a versatile epithelial model
Source: Pediatr Surg Int. 2026 Nov 13;43(1):18. doi: 10.1007/s00383-026-06704-z (PMC13768266; doi:10.1007/s00383-026-06704-z)
Supplement: Supplementary file 1 [file 383_2026_6704_MOESM1_ESM.docx]

*Supplementary Table 1. Patient samples details.*
